# Hormonal and reproductive factors and risk of nasopharyngeal carcinoma in Chinese women: a case-control study

**DOI:** 10.1186/s13104-025-07471-1

**Published:** 2025-11-14

**Authors:** Xiao-Ying Su, Axel Schroder, Lap Ah Tse, Ignatius Tak-sun Yu, Shao-Hua Xie

**Affiliations:** 1 School of Public Health, Fujian Medical University, Fuzhou, 350122 China; 2 Upper Gastrointestinal Surgery, Department of Molecular Medicine and Surgery, Karolinska Institutet, Blombäcks väg 23, 4th Floor, 17 177, Stockholm, Sweden; 3 Jockey Club School of Public Health and Primary Care, The Chinese University of Hong Kong, Hong Kong SAR, China; 4 Hong Kong Occupational and Environmental Health Academy, Hong Kong SAR, China

**Keywords:** Nasopharyngeal neoplasms, Gonadal steroid hormones, Reproductive history, Etiology, Risk factors

## Abstract

**Background:**

Sex hormones may play a role in the development of nasopharyngeal carcinoma (NPC), but the evidence is limited.

**Methods:**

This case-control study collected information on sex hormonal and reproductive factors of 99 female incident NPC cases and 109 female controls in Hong Kong through face-to-face interviews.

**Results:**

An increased risk of NPC associated with late menarche and spontaneous miscarriage history was indicated when adjusted for age only, the age-adjusted odds ratio (OR) were 2.22, 95% confidence interval (CI) 1.04–4.72 for menarche at ages > 14 years, 2.31 (95% CI 1.03–5.18) for one miscarriage, and 4.00 (95% CI 1.04–15.31) for ≥ 2 miscarriages. But such associations were attenuated after adjustment for additional potential confounders, the fully-adjusted OR were 1.78 (95% CI 0.76–4.14) for menarche at ages > 14 years, 2.11 (95% CI 0.90–4.99) for one miscarriage, and 3.42 (95% CI 0.76–15.32) for ≥ 2 miscarriages. No associations were found for the other studied exposures.

**Conclusions:**

Several hormonal exposures in relation to NPC risk were studied for the first time. However, this analysis led to inconclusive results given the small size but suggest no association between sex hormones and NPC in Hong Kong women.

**Supplementary Information:**

The online version contains supplementary material available at 10.1186/s13104-025-07471-1.

## Background

The incidence of nasopharyngeal carcinoma (NPC) varies greatly across geographical regions. It is rare in most parts of the world, but high incidence rates are noted in certain areas, including Southern China [1]. In 2021, the crude incidence rates of NPC were 16.5 and 5.3 per 100,000 in men and women, respectively, in Hong Kong [2].

NPC is more common in men than in women, and sex hormones may be involved in its development. A potential protective role of estrogens has been suggested by a delayed development of NPC among females, by around 5–10 years before menopause ages [3]. This hypothesis is also supported by potential anticancer properties of estrogen through mechanisms including inhibition of inflammation and initiating apoptosis in cancer cells [4]. However, a recent case-control study in Southern China reported an increased risk of NPC associated with a history of pregnancy or delivery in women [5]. The influence of sex hormonal exposures on the risk of NPC remains to be clarified.

This study aimed to assess the associations between sex hormonal and reproductive factors and NPC risk in Hong Kong women. We hypothesized that certain female sex hormonal exposures decrease the risk of NPC.

## Methods

This study included 99 female NPC patients and 109 female control participants in a case-control study conducted between June 2010 and December 2012 in Hong Kong. All newly diagnosed NPC patients with histological confirmation in Queen Elizabeth Hospital housing the largest oncology center in Hong Kong during the study period were recruited, and control participants were outpatients attending multiple clinical units of the same hospital and were frequency-matched by age (± 5 years) and residence district. Control participants were admitted to hospital for a wide range of health conditions. Considerable proportions of referents were diagnosed with diseases of the circulatory system (27%), diseases of the nervous system (13%), endocrine, nutritional and metabolic diseases (10.0%), and merely symptoms, signs and abnormal clinical and laboratory findings (12.7%). None of the other disease groups accounted for a proportion of 10% or more. Detailed information about the recruitment of participants can be found elsewhere [6, 7]. Information on the following sex hormonal exposures and reproductive factors were obtained through face-to-face interviews: age of menarche, menopause and first delivery, histories of pregnancies, deliveries and spontaneous miscarriage, and use of oral contraceptives and hormonal replacement therapy. The specific questions regarding these exposures are provided in the Appendix.

### Statistical analysis

Unconditional logistic regression was used to assess the associations between sex hormonal exposures and NPC risk, providing estimates for odds ratios (OR) and corresponding 95% confidence intervals (CI) with adjustment for age, education level, smoking status, family history of NPC, consumption of salted fish during childhood, and daily domestic incense burning. All statistical analysis was conducted by using SAS 9.4 software (SAS Institute Inco., Cary, NC) and a two-sided *P* < 0.05 was considered statistically significant.

## Results

The distribution of socioeconomic characteristics and main risk factors for NPC among cases and control participants is presented in Table 1.

**Table 1:** Distribution of social-demographic characteristics and main risk factors for nasopharyngeal carcinoma (NPC), number (%)

Variables	Cases (*N* = 99)	Controls (*N* = 109)
Age, years		
Mean (SD)	50.8 (± 9.3)	49.9 (± 11.1)
<45	25 (25.3)	36 (33.0)
45–51	41 (41.4)	39 (35.8)
≥55	33 (33.3)	34 (31.3)
Education level		
Primary school or lower	38 (38.4)	25 (22.9)
Middle school	52 (52.2)	53 (48.6)
Collage or higher	9 (9.1)	31 (28.4)
Employment status		
Unemployment	48 (48.5)	43 (29.5)
Employed	51 (51.5)	66 (66.6)
Married status		
Single	14 (14.1)	18 (16.5)
Married/cohabiting	70 (70.1)	77 (70.6)
Divorced/widowed/separated	13 (13.1)	13 (11.9)
Missing	2 (2.0)	1 (0.9)
Smoking status		
Non-smoker	84 (84.9)	99 (90.8)
Current smoker	10 (10.1)	9 (8.3)
Ex-smoker	5 (5.1)	1 (0.9)
Environmental tobacco smokes exposure		
No	24 (24.2)	39 (35.8)
At work or at home	66 (66.7)	63 (57.8)
Both	9 (9.1)	7 (6.4)
NPC family history in first relatives		
No	79 (79.8)	104 (95.4)
Yes	20 (20.2)	5 (4.6)
Salted fish intake during childhood		
< Monthly	38 (38.4)	51 (46.8)
Monthly	27 (27.3)	34 (31.2)
At least weekly	30 (30.3)	19 (17.4)
Missing	4 (4.0)	5 (4.6)
Self-reported occupational exposure*	31 (31.3)	15 (13.8)
Daily domestic burning incense	64 (64.7)	47 (43.1)

^*^Exposures to any of cotton dust, chemical fumes, welding fumes and disinfectants

Menarche at age of 14 years or later was associated with an increased risk of NPC when adjusted for age only (OR = 2.22, 95% CI 1.04–4.72), but such association was statistically insignificant after adjustments for additional covariates (OR = 1.78, 95% CI 0.76–4.14). History of spontaneous miscarriage was associated with an increased risk of NPC. The age-adjusted OR were 2.31 (95% CI 1.03–5.18) for one miscarriage and 4.00 (95% CI 1.04–15.31) for two or more miscarriages, but such associations were attenuated with further adjustment (OR = 2.11, 95% CI 0.90–4.99 for one miscarriage; OR = 3.42, 95% CI 0.76–15.32 for two or more miscarriages; *P* for trend 0.0274). No associations were found for the other studied exposures, including menstrual cycle regularity, menopausal status, use of oral contraceptives, number of pregnancies and deliveries, and age at first delivery (Table 2).

**Table 2:** Associations between sex hormonal and reproductive factors and risk of nasopharyngeal carcinoma

Exposures	Cases (*N* = 99)	Controls (*N* = 109)	Age-adjusted OR (95% CI)	Fully adjusted OR^*^ (95% CI)
Age of menarche, years				
<13	33 (33.3)	41 (37.6)	1.00 (Reference)	1.00 (Reference)
13–14	24 (24.2)	40 (36.7)	0.75 (0.38–1.50)	0.78 (0.36–1.66)
>14	33 (33.3)	19 (17.4)	2.22 (1.04–4.72)	1.78 (0.76–4.14)
Missing	9 (9.1)	9 (8.3)		
P for trend			0.0657	0.2559
Menstrual cycle regularity				
Regular	12 (12.1)	15 (13.8)	1.00 (Reference)	1.00 (Reference)
Irregular	80 (80.1)	90 (82.6)	0.92 (0.40–2.13)	0.66 (0.26–1.69)
Missing	7 (7.1)	4 (3.7)		
Postmenopausal status				
No	51 (51.5)	51 (46.8)	1.00 (Reference)	1.00 (Reference)
Yes	41 (41.4)	45(41.2)	0.79 (0.33–1.90)	0.54 (0.19–1.50)
Missing	7 (7.1)	13 (12.0)		
Ever pregnancy				
No	20 (20.2)	21 (19.3)	1.00 (Reference)	1.00 (Reference)
Yes	79 (79.8)	88 (80.7)	0.90 (0.45–1.81)	0.81 (0.36–1.81)
Number of pregnancies				
0	20 (20.2)	21 (19.3)	1.37 (0.65–2.90)	1.40 (0.60–3.30)
1–2	36 (36.4)	52 (47.7)	1.00 (Reference)	1.00 (Reference)
≥3	41 (41.4)	33 (30.3)	1.82 (0.93–3.58)	1.58 (0.74–3.39)
Missing	2 (2.0)	3 (2.8)		
P for trend			0.3889	0.6932
Number of deliveries				
0	25 (25.3)	23 (21.1)	1.40 (0.71–2.79)	1.48 (0.67–3.25)
1–2	49 (49.5)	61 (56.0)	1.00 (Reference)	1.00 (Reference)
≥3	22 (22.2)	23 (21.1)	1.09 (0.53–2.28)	0.86 (0.37–1.97)
Missing	3 (3.0)	2 (1.8)		
P for trend			0.5444	0.2941
Age at first delivery				
No	20 (20.2)	21 (19.2)	1.00 (Reference)	1.00 (Reference)
≤27	47 (47.5)	44 (40.4)	1.10 (0.51–2.40)	0.89(0.36–2.22)
>27	24 (24.2)	39 (35.8)	0.64 (0.29–1.43)	0.64 (0.26–1.58)
Missing	8 (8.1)	5 (4.6)		
P for trend			0.2069	0.3051
Number of spontaneous miscarriages				
0	69 (69.7)	93 (85.3)	1.00 (Reference)	1.00 (Reference)
1	19 (19.2)	11 (10.1)	2.31 (1.03–5.18)	2.11 (0.90–4.99)
≥2	9 (9.1)	3 (2.8)	4.00 (1.04–15.31)	3.42 (0.76–15.32)
Missing	2 (2.0)	2 (1.8)		
P for trend			0.007	0.0274
Ever use of oral contraceptives				
No	61 (61.6)	70 (64.2)	1.00 (Reference)	1.00 (Reference)
Yes	32 (32.3)	34 (31.2)	1.07 (0.59–1.94)	1.03 (0.53-2.00)
Missing	6 (6.1)	5 (4.6)		

^*^Adjusted for age, education level, smoking status, family history of nasopharyngeal carcinoma, consumption of salted fish during childhood, and daily domestic incense burning

## Discussion

Epidemiological evidence regarding the role of sex hormones in NPC has been sparse. To our knowledge, the associations between reproductive factors and NPC risk have been reported in a previous case-control study only [5]. For the first time, the present study investigated several sex hormonal exposures including age of menarche and first delivery, menstrual cycle regularity, history of spontaneous miscarriage, and use of oral contraceptives and hormonal replacement therapy. Overall, no associations were found between the studied exposures and NPC risk in women. A seemingly increased NPC risk associated with spontaneous miscarriage was noted, which however may be explained by residual confounding or chance.

Limitations of the present study mainly include the hospital-based design due to practical difficulties, retrospectively collected information on exposures, no adjustment for Epstein-Barr virus infection, and the limited sample size due to the rarity of NPC particularly in women, with *post hoc* estimation of statistical power ranging 0.09–0.54 only. Exposure misclassification was probably non-differential because sex hormonal exposures are not established risk factors of NPC and participants were unaware of the study hypothesis.

An early cross-sectional analysis of 14 Kenyan men with NPC reported substantially low levels of dehydroepiandrosterone-sulphate in Kenyan men with NPC compared with control participants [8], but no prospective studies have direct assessed endogenous sex hormone levels in relation to NPC risk.

In summary, our findings do not support the hypothesis that sex hormonal exposures influence the risk of NPC in women. More epidemiological studies are warranted to further clarify the role of sex hormonal exposures in the development of NPC.

## Supplementary Information

Below is the link to the electronic supplementary material.

Supplementary Material 1.

## Data Availability

Data supported the conclusion of this study was included in this manuscript. Other data used in this research are not publicly due to privacy and the potential benefits and risks, but emails could be sent to the corresponding author (email: shaohua.xie@ki.se) for data sharing upon request.

## Abbreviations

NPC: Nnasopharyngeal carcinoma
OR: Odds ratio
CI: Confidence interval
SD: Standard deviation

## Declarations

### Provenance and peer review

This article was not commissioned and was externally peer reviewed.
