# Supplementary material for: Hormonal and reproductive factors and risk of nasopharyngeal carcinoma in Chinese women: a case-control study
Source: BMC Res Notes. 2025 Nov 14;18:483. doi: 10.1186/s13104-025-07471-1 (PMC12619248; doi:10.1186/s13104-025-07471-1)
Supplement: Supplementary file 1 — Supplementary Material 1. [file 13104_2025_7471_MOESM1_ESM.pdf]

Appendix: Questions regarding reproductive factors and hormonal exposures

(Translated from Chinese)

This section on women's health-related issues is for female respondents only.

Finally, we would like to ask you a few questions regarding women's health. These questions are extremely valuable for understanding the current health situation of women in Hong Kong. There are only a few very simple questions.

Menstrual history:

1. How old were you when you had your first period (menarche)?
2. Is your period usually regular?

   ☐ 1 Regular ☐ 2 Irregular

   (If you have already reached menopause, please answer based on your situation before menopause.)
3. When was your last period (most recent menstrual cycle)? Year / Month

   If you have reached menopause (no periods for one year or longer):

   Have you taken any hormone-based medications after menopause?

   ☐ 0 No ☐ 1 Yes

Have you ever been pregnant?

☐ 1 Yes ☐ 0 No

If yes: Number of pregnancies: ___ Number of births: ___

If the number of births is less than the number of pregnancies, please indicate:

Number of natural miscarriages: ___

If you have given birth, how old were you at the time of your first birth? ___ years old

Have you ever used contraceptives in the past?

☐ 0 No ☐ 1 Yes
